# Right heart size and function significantly correlate in patients with pulmonary arterial hypertension – a cross-sectional study

**DOI:** 10.1186/s12931-018-0913-x

**Published:** 2018-11-08

**Authors:** Lukas Fischer, Nicola Benjamin, Norbert Blank, Benjamin Egenlauf, Christine Fischer, Satenik Harutyunova, Maria Koegler, Hanns-Martin Lorenz, Alberto M. Marra, Christian Nagel, Panagiota Xanthouli, Eduardo Bossone, Ekkehard Grünig

**Affiliations:** 1 Centre for Pulmonary Hypertension, Thoraxklinik at Heidelberg University Hospital, Röntgenstrasse 1, D-69126 Heidelberg, Germany; 2 Translational Lung Research Center Heidelberg (TLRC), Member of the German Center for Lung Research (DZL), Heidelberg, Germany; 3 Department of Rheumatology, University Hospital Heidelberg, Heidelberg, Germany; 4 Institute of Human Genetics, University of Heidelberg, Heidelberg, Germany; 5 IRCCS SDN Research Institute, Naples, Italy; 6 Lung Centre, Klinikum Mittelbaden, Baden-Baden Balg, Baden-Baden, Germany; 7 Heart Department, Cardiology Division, “Cava de’ Tirreni and Amalfi Coast” Hospital, University of Salerno, Salerno, Italy

**Keywords:** Pulmonary hypertension, Right ventricular output reserve, Pump function, Right ventricular size, Right atrial size

## Abstract

**Background:**

The objective of this study was to assess, whether right atrial (RA) and ventricular (RV) size is related to RV pump function at rest and during exercise in patients with pulmonary arterial hypertension (PAH).

**Methods:**

We included 54 patients with invasively diagnosed PAH that had been stable on targeted medication. All patients underwent clinical assessments including right heart catheterization and echocardiography at rest and during exercise. RV output reserve was defined as increase of cardiac index (CI) from rest to peak exercise (∆CI_exercise_). Patients were classified according to the median of RA and RV-area. RV pump function and further clinical parameters were compared between groups by student’s t-test. Uni- and multivariate Pearson correlation analyses were performed.

**Results:**

Patients with larger RA and/or RV-areas (above a median of 16 and 20cm^2^, respectively) showed significantly lower ∆CI_exercise_**,** higher mean pulmonary arterial pressure, pulmonary vascular resistance at rest and NT-proBNP levels. Furthermore, patients with higher RV-areas presented with a significantly lower RV stroke volume and pulmonary arterial compliance at peak exercise than patients with smaller RV-size. RV area was identified as the only independent predictor of RV output reserve.

**Conclusion:**

RV and RA areas represent valuable and easily accessible indicators of RV pump function at rest and during exercise. Cardiac output reserve should be considered as an important clinical parameter. Prospective studies are needed for further evaluation.

## Background

Pulmonary arterial hypertension (PAH) is a complex cardiopulmonary disorder, characterized by progressive changes affecting both the pulmonary vasculature and the right heart [1, 2]. Although the initial pathological changes occur on pulmonary arterioles causing increased pulmonary vascular resistance (PVR), adaptation of right ventricular (RV) pump function is a key determinant of survival [2, 3]. Rising attention is drawn to the concept of RV-arterial coupling, a composite measure of RV pump function and ventricular load [4–6].

Right atrial (RA) [7–9] and RV size have repeatedly been proven of prognostic significance in pulmonary hypertension [2, 10], whereas their impact on RV contractility remains to be determined. Recent studies using magnetic resonance imaging (MRI) have shown, that increased RV-endsystolic or diastolic volumes were significantly related to a worse outcome and reduced RV stroke volume (SV) [11]. In a further study enlargement of RV volumes during follow-up was associated with further clinical signs of disease progression [12].

RV output reserve (∆CI_exercise_) defined as increase of cardiac output/cardiac index (CI) during exercise with normal or elevated PVR measured by right heart catheterization (RHC) is an emerging parameter which has shown to be prognostically important in patients with PAH [13, 14]. It solely displays the capacity of the right ventricle to adjust its systolic function to a given level of pulmonary loading^4^. Pulmonary arterial compliance (PAC) reflects the elasticity of the pulmonary arteries. For estimation of pulmonary arterial compliance (or capacitance) the measurement of SV/pulse pressure (cardiac output/heart rate)/(systolicPAP-diastolicPAP) by RHC has been shown to be the most simple and practical method [15, 16].

The objective of the study was to investigate the correlation between right heart size (measured as right atrial and ventricular area by echocardiography) and RV pump function at rest and during exercise (assessed by RHC) and further hemodynamic and clinical parameters. Furthermore, this study aimed to detect correlations and determining factors of RV pump function.

## Methods

### Patient selection

We retrospectively reviewed all incident (i.e. newly diagnosed) patients aged ≥18 to 80 years with idiopathic, heritable or drug- and toxin-induced or connective tissue disease associated PAH who were diagnosed at the PH-center in Heidelberg between January 1st, 2016 and November 31st, 2016. Inclusion required RHC at rest (confirming PAH, defined as a mean pulmonary arterial pressure ⩾25 mmHg, pulmonary arterial wedge pressure ⩽15 mmHg and PVR > 3 Wood units [17], and during exercise. Diagnosis of PAH was performed according to the ESC/ERS guidelines [17].

Patients were excluded if they lacked a complete evaluation including medical history, WHO/NYHA functional class assessment, physical examination, electrocardiogram, transthoracic 2D-echocardiography at rest, lung function test, arterial blood gases, 6-min walking distance (6MWD) under standardized conditions [18], laboratory testing including NT-proBNP levels. All examinations were performed at the Thoraxklinik at Heidelberg University Hospital by experienced physicians within 48 h from the right heart catheterization.

#### Right heart catheterization

The hemodynamic values have been obtained by the charts. The right heart catheterization has been performed in a standardized way in a supine position using the transjugular access with a triple-lumen 7F-Swan-Ganz thermodilution catheter at rest and during exercise as previously described [19]. Patients had been examined on a variable load supine bicycle ergometer by experienced investigators (CN, BE, SH). Pressures were continuously recorded and averaged over several respiratory cycles during spontaneous breathing, both at rest and during exercise. Cardiac output (CO) was measured by thermodilution at least in triplicate with a variation of less than 10% between the measured values. The zero reference point for pressure recordings was set at ½ of the thoracic diameter below the anterior thorax surface [20]. After the hemodynamic measurement at rest, the supine position was changed to a 45° position. Calibration for exercise measurements were performed as previously described [21]. The exercise test was started with a workload of 25 W. Workload was incrementally increased by 25 W every 2 min to an exercise capacity or symptom limited maximum.

#### Echocardiography

Resting two dimensional transthoracic echocardiography (TTE) Doppler examinations were performed by experienced cardiac sonographers (EG, CN, BE, SH) with commercially available equipment (Vivid 7, GE Healthcare, Milwaukee, Wisconsin) according to standardized protocol as described previously [9, 22]. TTE measurements were obtained off line from stored DICOM data according to the European Association of Cardiovascular Imaging (EACVI) Guidelines [23]. Specific indices included RA-/RV-area, TAPSE and PASP at rest. For all calculations the mean value of at least 3 measurements was used. PASP was estimated from peak tricuspid regurgitation jet velocities (TRV) according to the equation: PASP = 4 (V) [2] + right atrial pressure, where V is the peak velocity (in m/s) of tricuspid regurgitation jet (TRV) [24]. Right atrial pressure was estimated from characteristics of the inferior vena cava [18]. If it was < 20 mm in diameter and decreased during inspiration we added 5 mmHg, ≥20 mm we added 10 mmHg and 15 mmHg if no decrease of diameter during inspiration occurred.

#### Cardiopulmonary exercise testing

Patients were examined on a variable load supine bicycle ergometer (model 8420; KHL Corp., Kirkland, Washington) in Heidelberg as described previously [25]. Workload was increased by 25 W every 2 min to an exercise capacity or symptom limited maximum. Peak VO_2_ was defined as the highest 30-s average value of oxygen uptake during the last minute of the exercise test.

### Ethics statement

The Ethics Committee of the Medical Faculty, University of Heidelberg had no obligation against the conduct of the study (internal number S425/2016). All data were anonymized and the study was conducted in accordance with the amended Declaration of Helsinki.

### Statistical methods

Statistical analyses were conducted by two biometricians (CF, NE). Data are described as means ± standard deviations or number and respective percentage. Patients were divided into two groups according to their RV size (larger or smaller RA and/or RV area with value above or below the median of the complete sample). A receiver operating characteristic (ROC) curve analysis for RA and RV area with CI increase below the median of the sample as outcome parameter for further validation of the cut-off values was performed. Quantitative characteristics between the two groups including demographics, hemodynamics and parameters of echocardiography and cardiopulmonary exercise testing were compared by two-sided student’s t-tests and nonparametric tests if needed. Frequency distributions were compared by chi-square test or Fisher’s exact test. A sensitivity analysis with a threshold of 18 cm^2^ for RV area according to the cut-off proposed by the guidelines^17^ was performed.

Right heart size (RA and RV area) was compared between patients with higher vs. lower ∆CI_exercise_ (according to the median of the complete sample).

Differences of the course of CI and SV increase during exercise between patients with smaller vs. larger RA and RV area were analysed with mixed ANOVA. To investigate the associations between clinical parameters, right heart size and output reserve, Pearson’s correlation analysis was performed. To identify independent predictors of RV output reserve, multivariate analysis was performed by stepwise forward selection method of logistic regression with the dichotomous variable of the two groups (high or low ∆CI_exercise_) as outcome variable. Parameters for correlation analysis included demographics, hemodynamics, echocardiographic parameters and measures of cardiopulmonary exercise testing according to clinical significance.

Pulmonary arterial compliance (PAC) was calculated according the formula PAC = SV/ pulse pressure with SV = CO/Heart rate and pulse pressure = sPAP-dPAP. Stroke volume index was calculated with SVI = CI / heart rate.

All tests were two-sided and a pointwise *p*-value of 0.05 was considered statistically significant. All analyses have been performed using IBM SPSS 23 (SPSS Statistics V23, IBM Corporation, Somers, New York).

## Results

### Study population (Table 1)

We included 54 patients diagnosed with moderate to severe PAH who fulfilled the inclusion criteria (21 males and 33 females, mean age 53 ± 15 years, 66.7% WHO functional class II, 57.4% double combination therapy; Table 1).

**Table 1:** Characteristics of the study population

				mean ± SD or n (%)		
Demographics		Age	*(years)*	53	±	14.65
		BMI	*(kg/m* ^*2*^ *)*	27.9	±	5.69
Gender		male	*n (%)*	21		(38.9)
		female	*n (%)*	33		(61.1)
Diagnosis		IPAH	*n (%)*	31		(57.4)
		HPAH	*n (%)*	8		(14.8)
		APAH	*n (%)*	12		(22.2)
		CTEPH	*n (%)*	3		(5.6)
WHO functional class		I	*n (%)*	1		(1.9)
		II	*n (%)*	36		(66.7)
		III	*n (%)*	17		(31.5)
PAH-targeted medication		Endothelin receptor antagonist		40		(74.1)
		Phosphodiesterase-5-inhibitors		38		(70.4)
		Soluble guanylate cyclase-stimulator		8		(13.0)
		Prostanoids		6		(14.8)
		Calcium channel blockers		2		(03.7)
Combination therapy						
		Mono	*n (%)*	18		(33.3)
		Double	*n (%)*	31		(57.4)
		Triple	*n (%)*	5		(9.3)
RHC	Rest	mPAP	*(mmHg)*	35.5	±	11.69
		sPAP	*(mmHg)*	57.6	±	20.87
		dPAP	*(mmHg)*	23	±	7.87
		PCWP	*(mmHg)*	10	±	3.54
		PVR	(dyn*sec*cm ^*−5*^ *)*	393.4	±	235.03
		CO	*(l/min)*	5.8	±	1.61
		CI	*(l/min/m* ^*2*^ *)*	3	±	0.73
		SVI	*(ml/m* ^*2*^ *)*	41.1	±	10.2
	25 W	∆ CI	*(l/min/m* ^*2*^ *)*	1.2	±	0.67
	50 W	∆ CI	*(l/min/m* ^*2*^ *)*	2	±	0.93
	75 W	∆ CI	*(l/min/m* ^*2*^ *)*	2.6	±	1.15
	Peak	mPAP	*(mmHg)*	56.5	±	15.91
		sPAP	*(mmHg)*	90.2	±	28.36
		dPAP	*(mmHg)*	36.1	±	11.56
		CO	*(l/min)*	10.2	±	3.49
		CI	*(l/min/m* ^*2*^ *)*	5.3	±	1.59
		SVI	*(ml/m* ^*2*^ *)*	47.2	±	13.9
Echocardiography		RV area	*(cm* ^*2*^ *)*	20.1	±	5.59
		RA area	*(cm* ^*2*^ *)*	16.8	±	6.62
		TAPSE	*(cm)*	2.3	±	0.38
Cardiopulmonary exercise testing (CPET)		peak V’O_2_	*(ml/min)*	1126	±	428.88
		peak V’O_2_/kg	*(ml/min/kg)*	14.1	±	3.92
		sPAP _Max_	*(mmHg)*	81.8	±	27.87
		6-MWD	*(m)*	423	±	113.09
Laboratory analysis		NT-proBNP	*(pg/ml)*	470.3	±	856.74
Pulmonary function test (PFT)		DLCOc SB	*(% Soll)*	58.6	±	17.25
		DLCOc VA	*(% Soll)*	70.42	±	20.00

*IPAH* = idiopathic pulmonary arterial hypertension, *HPAH* = heritable PAH, *APAH* = associated PAH, CTEPH = chronic thromboembolic PH, *RHC* = right heart catheter, *BMI* = Body Mass Index, *RV* = right ventricular, RA = right atrial, TAPSE = tricuspid annular plane systolic excursion, *VO’*_*2*_ = oxygen consumption, *NT-proBNP* = N-terminal pro brain natriuretic peptide, *DLCOc SB* = diffusing capacity transfer factor, *DLCOc / VA* = diffusing capacity transfer coefficient, mPAP = mean pulmonary arterial pressure, *sPAP* = systolic PAP, *dPAP* = diastolic PAP, *PCWP* = pulmonary capillary wedge pressure, *PVR* = pulmonary vascular resistance, CI = Cardiac Index, *SVI* = stroke volume index, *HR* = heart rate, *SV* = stroke volume, ∆ = difference

**Fig. 1:**
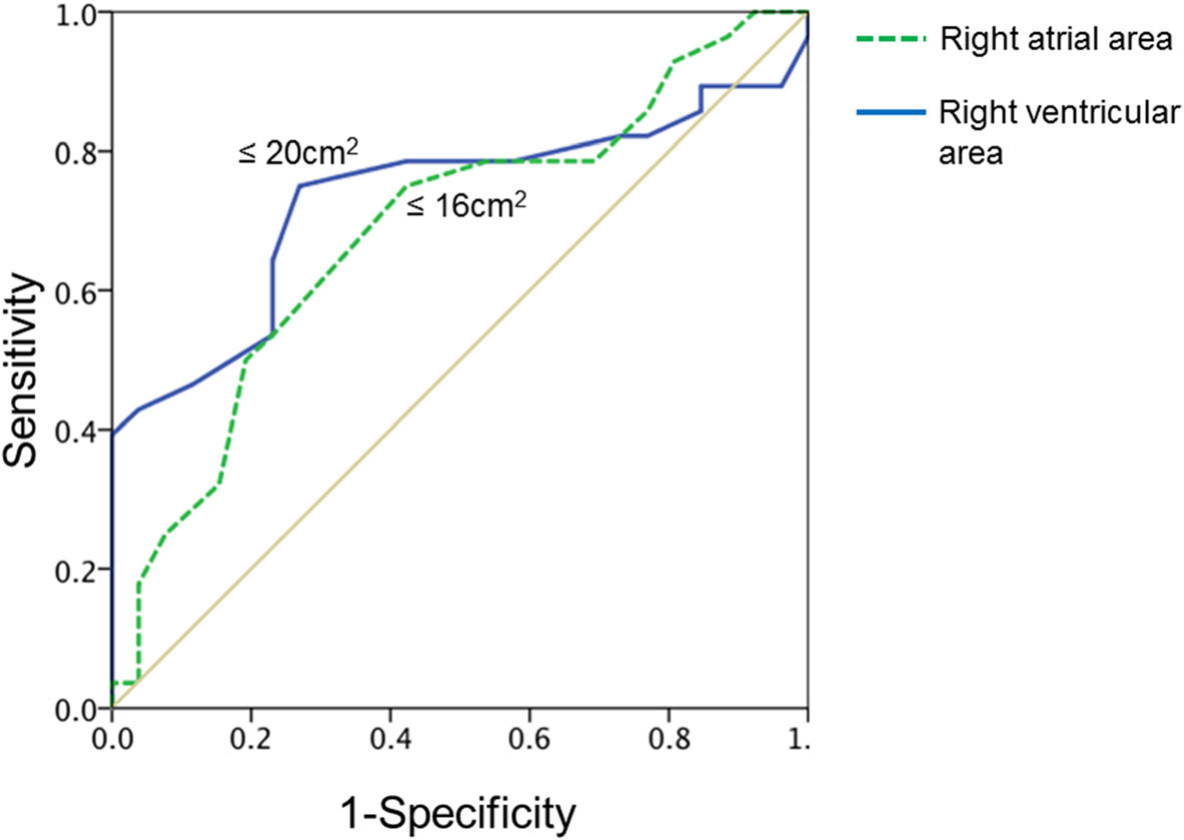
ROC curve analysis. For RV area, 20cm^2^ showed a sensitivity of 75% and specificity of 73.1%; an RA area of 16cm^2^ presented with a sensitivity of 75% and specificity of 57.7%

The study cohort presented with a median RA of 16cm^2^ and RV of 20 cm^2^. ROC curve analysis for RA and RV area with CI increase < 2.1 l/min/m^2^ (median of the sample for CI increase) further supported these proposed cutoff-values of 16cm^2^ for RA and 20cm^2^ for RV area (Fig. 1). For RV area, 20cm^2^ showed a sensitivity of 75% and specificity of 73.1%; an RA area of 16cm^2^ presented with a sensitivity of 75% and specificity of 57.7%.

**Characteristics of groups with small and large right heart size:** According to the median RA and RV area, two subgroups were defined for both RA and RV area: **1) “enlarged right heart size”** (RA >16cm^2^, RV >20cm^2^) and **2) “normal/smaller right heart size”** (RA ≤16cm^2^, RV ≤20cm^2^; Table 2).

**Table 2:** Comparison of patients with small and large right heart size

				n	RA area ≤ 16 cm^2^			n	RA area > 16 cm^2^			p-value		n	RV area ≤ 20 cm^2^			n	RV area > 20 cm^2^			p-value	
Demographics		Age	*(years)*	33	53.6	±	14.4	21	52.1	±	15.3	0.71		30	51.4	±	13.7	24	55.1	±	15.8	0.354	
		BMI	*(kg/m* ^*2*^ *)*	33	28.3	±	6.5	21	27.3	±	4.3	0.537		30	27.5	±	5.5	24	28.4	±	6.1	0.591	
		6-MWD	*(m)*	32	429	±	124	21	414	±	97	0.655		30	436	±	127	23	406	±	92	0.350	
PAH-targeted medication		ERA			24	(72.7%)			16	(76.2%)		1.0			22	(73.3%)			18	(75%)		1.0	
		PDE5-I			22	(66.7%)			16	(76.2%)		0.549			21	(70%)			17	(70.8%)		1.0	
		sGC stimulator			3	(9.1%)			5	(23.8%)		0.238			3	(10%)			5	(20.8%)		0.443	
		Prostanoids			3	(9.1%)			3	(14.3%)		0.667			2	(6.7%)			4	(16.7%)		0.389	
		Calcium channel blockers			2	(6.1%)			0			0.516			2	(6.7%)			0			0.497	
Combination therapy		Mono	*n (%)*		13	(39.4%)			5	(23.8%)		0.102			11	(36.7%)			7	(29.2%)		0.414	
		Double	*n (%)*		17	(51.5%)			14	(66.7%)					17	(56.7%)			14	(58.3%)			
		Triple	*n (%)*		3	(9.1%)			2	(9.5%)					2	(6.6%)			3	(12.5%)			
Echocardiography		RV area	*(cm* ^*2*^ *)*	33	17.3	±	3.9	21	24.6	±	4.9	< 0.0001	*	30	16.3	±	3.1	24	25.0	±	4.0	< 0.0001	*
		RA area	*(cm* ^*2*^ *)*	33	13.2	±	2.7	21	22.4	±	7.1	< 0.0001	*	30	13.3	±	3.2	24	21.1	±	7.2	< 0.0001	*
		sPAP	*(mmHg)*	33	42	±	16	21	57	±	17	0.003	*	30	42	±	12	24	56		20	0.006	*
		TAPSE	*(cm)*	33	2.4	±	0.4	21	2.3	±	0.4	0.362		30	2.4	±	0.4	24	2.3	±	0.4	0.204	
Cardiopulmonary Exercise Testing		peak V’O_2_	*(ml/min)*	33	1155	±	495	21	1080	±	302	0.536		30	1144	±	435	24	1103	±	429	0.730	
		peak V’O_2_/kg	*(ml/min/kg)*	33	14.5	±	4.1	21	13.4	±	3.7	0.321		30	14.7	±	3.6	24	13.2	±	4.2	0.166	
Laboratory analysis		NT-proBNP	*(pg/ml)*	33	191	±	231	20	931	±	1266	0.018	*	30	145	±	125	23	895	±	1191	0.006	*
Pulmonary function test		DLCOc SB	*(% Soll)*	30	58.6	±	18.6	18	58.6	±	15.2	0.988		27	60.5	±	15.3	21	56.2	±	19.6	0.398	
		DLCOc VA	*(% Soll)*	30	69.0	±	21.2	18	72.8	±	18.1	0.522		27	70.1	±	19.3	21	70.8	±	21.3	0.906	
RHC	Rest	mPAP	*(mmHg)*	33	32	±	10	21	41	±	13	0.012	*	30	32	±	9	24	40	±	13	0.014	*
		sPAP	*(mmHg)*	33	51	±	17	21	68	±	22	0.002	*	30	51	±	16	24	66	±	24	0.013	*
		dPAP	*(mmHg)*	33	21	±	6	21	26	±	9	0.032	*	30	21	±	6	24	26	±	9	0.018	*
		PAWP	*(mmHg)*	33	10	±	3	21	10	±	4	0.514		30	10	±	3	24	10	±	4	0.852	
		PVR	(dyn*sec*cm ^*− 5*^ *)*	33	335	±	201	21	486	±	259	0.03	*	30	311	±	149	24	496	±	282	0.006	*
		CI	*(l/min/m* ^*2*^ *)*	33	3.08	±	0.63	21	2.93	±	0.88	0.470		30	3.19	±	0.67	24	2.82	±	0.77	0.062	
		HR	*(1/min)*	33	74	±	12	21	76	±	12	0.480		30	75	±	10	24	75	±	13	0.927	
		SV	*(ml)*	33	79.4	±	21.2	21	77.1	±	25.4	0.716		30	80.1	±	19.0	24	76.4	±	27.0	0.556	
		SVI	*(ml/m* ^*2*^ *)*	33	42.2	±	8.4	21	39.3	±	12.6	0.317		30	42.9	±	8.4	24	38.8	±	11.9	0.142	
	25 W	∆ CI	*(l/min/m* ^*2*^ *)*	31	1.4	±	0.7	20	0.9	±	0.5	0.008	*	28	1.3	±	0.7	23	1.0	±	0.6	0.114	
		HR	*(1/min)*	31	91	±	18	20	93	±	12	0.572		28	91	±	18	23	93	±	13	0.623	
		∆ SV	*(ml)*	31	14.6	±	20.4	20	6.2	±	12.3	0.105		28	15.0	±	21.5	23	6.9	±	11.5	0.112	
	50 W	∆ CI	*(l/min/m* ^*2*^ *)*	31	2.2	±	1.0	18	1.6	±	0.7	0.027	*	28	2.28	±	0.91	21	1.51	±	0.76	0.003	*
		HR	*(1/min)*	31	103	±	17	18	105	±	17	0.639		28	102	±	18	21	106	±	15	0.345	
		∆ SV	*(ml)*	31	18.0	±	22.7	18	7.2	±	13.1	0.041	*	28	21.0	±	22.5	21	4.7	±	12.0	0.002	*
	75 W	∆ CI	*(l/min/m* ^*2*^ *)*	18	2.9	±	1.2	9	2.0	±	0.6	0.043	*	16	2.93	±	1.17	11	2.09	±	0.96	0.060	
		HR	*(1/min)*	18	113	±	16	9	112	±	12	0.813		16	111	±	17	11	115	±	10	0.541	
		∆ SV	*(ml)*	18	22.2	±	18.1	9	7.4	±	18.1	0.045	*	16	25.0	±	14.2	11	6.0	±	18.2	0.005	*
	Peak	mPAP	*(mmHg)*	33	54	±	15	21	61	±	17	0.115		30	54	±	15	24	60	±	17	0.124	
		sPAP	*(mmHg)*	33	84	±	26	21	100	±	30	0.049	*	30	85	±	25	24	97	±	31	0.144	
		dPAP	*(mmHg)*	33	35	±	12	21	38	±	11	0.254		30	34	±	12	24	39	±	10	0.111	
		CI	*(l/min/m* ^*2*^ *)*	33	5.62	±	1.57	21	4.80	±	1.52	0.049	*	30	5.92	±	1.43	24	4.52	±	1.45	0.001	*
		∆ CI	*(l/min/m* ^*2*^ *)*	33	2.54	±	1.42	21	1.86	±	0.83	0.033	*	30	2.73	±	1.34	24	1.7	±	0.88	0.002	*
		SV	*(ml)*	33	93.3	±	26.7	21	86.0	±	33.8	0.383		30	0.1	±	0.027	24	0.08	±	0.029	0.007	*
		PAC	*(ml/mmHg)*	33	39.0	±	13.7	21	33.1	±	12.4	0.108		30	39.5	±	11.2	24	33.2	±	15.3	0.027	*
		SVI	(l/m^2^)	33	49.4	±	11.5	21	43.7	±	16.6	0.141		30	53.1	±	11.1	24	39.8	±	13.7	< 0.001	*

*ERA* = Endothelin receptor antagonist, *PDE5-I* = Phosphodiesterase-5-inhibitors, *sGC stimulator* = Soluble guanylate cyclase-stimulator, *RHC* = right heart catheter, *BMI* = Body Mass Index, *RV* = right ventricular, *RA* = right atrial, *TAPSE* = tricuspid annular plane systolic excursion, *VO’2* = oxygen consumption, *NT-proBNP* = N-terminal pro brain natriuretic peptide, *DLCOc SB* = diffusion capacity transfer factor, *DLCOc / VA* = diffusion capacity transfer coefficient, *mPAP* = mean pulmonary arterial pressure, *sPAP* = systolic PAP, *dPAP* = diastolic PAP, *PAWP* = pulmonary arterial wedge pressure, *PVR* = pulmonary vascular resistance, *CI* = Cardiac Index, *SVI* = stroke volume index, *HR* = heart rate, *SV* = stroke volume, *∆* = difference

* = significant at level 0.05.; values are given as mean ± standard deviation or n (%)

Both groups did not significantly differ in their demographics (age and BMI), 6MWD, diffusion capacity and peak VO_2_ for both RA and RV area. PH-targeted treatment and distribution of combination treatment did also not significantly differ between groups (Table 2).

Patients with enlarged RA- (*n* = 21) and/or RV-area (*n* = 24) had significantly higher mean, systolic and diastolic pulmonary arterial pressures, mean pulmonary vascular resistance, and NT-proBNP levels than patients with normal or smaller right heart size.

**Fig. 2:**
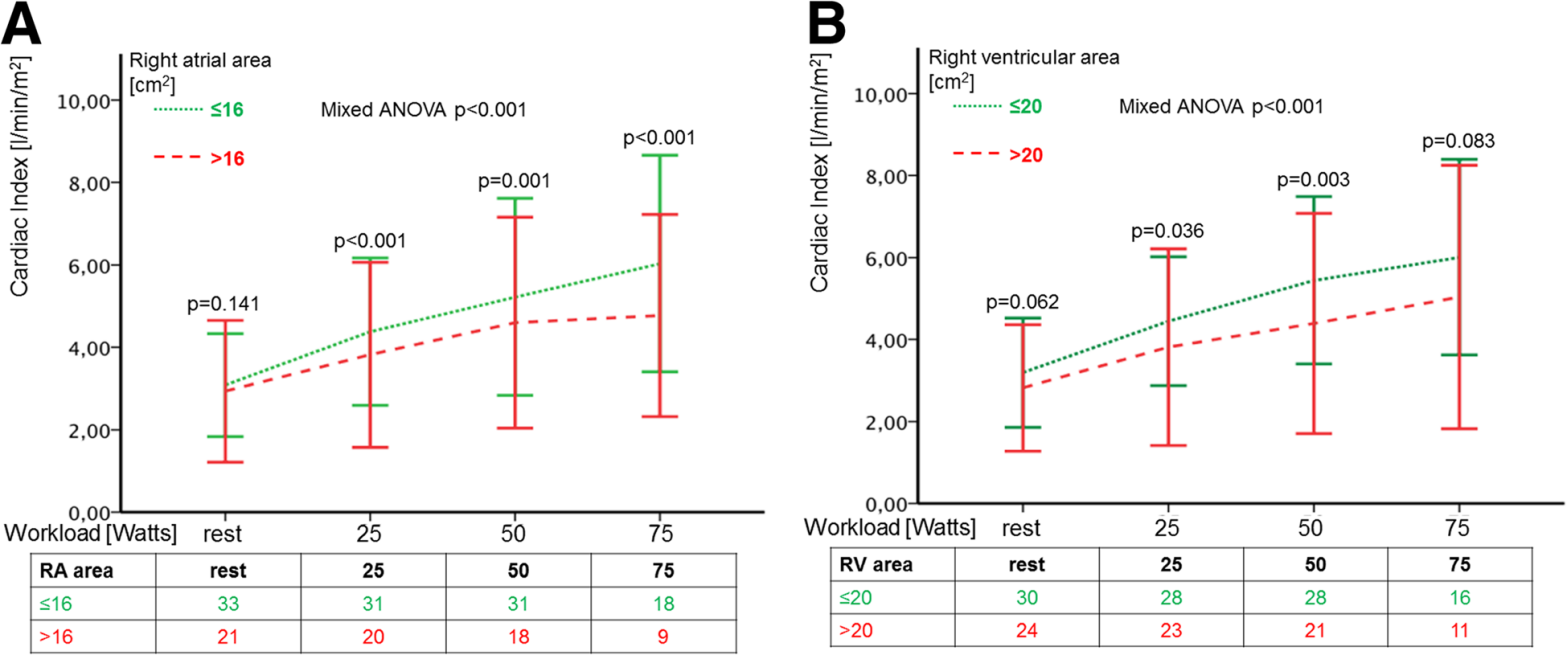
Course of CI during exercise according to RA (**a**) and RV area (**b**). Patients with smaller (RA ≤ median 16 cm^2^, RV ≤ median 20 cm^2^, dotted line) right heart size showed significantly higher CI during exercise, than patients with larger right heart size (RA > median 16 cm^2^, RV > median 20 cm^2^, dashed line; mixed ANOVA *p* < 0.001). Bars indicate 2 standard deviations of the mean

Both groups of RA and RV size had well preserved RV function at rest, represented by regular CI and SV, even though PVR and mean pulmonary arterial pressures were elevated in patients with enlarged right heart size. Increase of CI during exercise was significantly smaller in patients with enlarged RA- or RV-areas (Fig. 2a and b). Furthermore, patients with higher RV-area, but not RA-area, presented with a significantly lower SV, SVI and pulmonary arterial compliance at peak exercise than patients with smaller RV-size (Table 2).

**Fig. 3:**
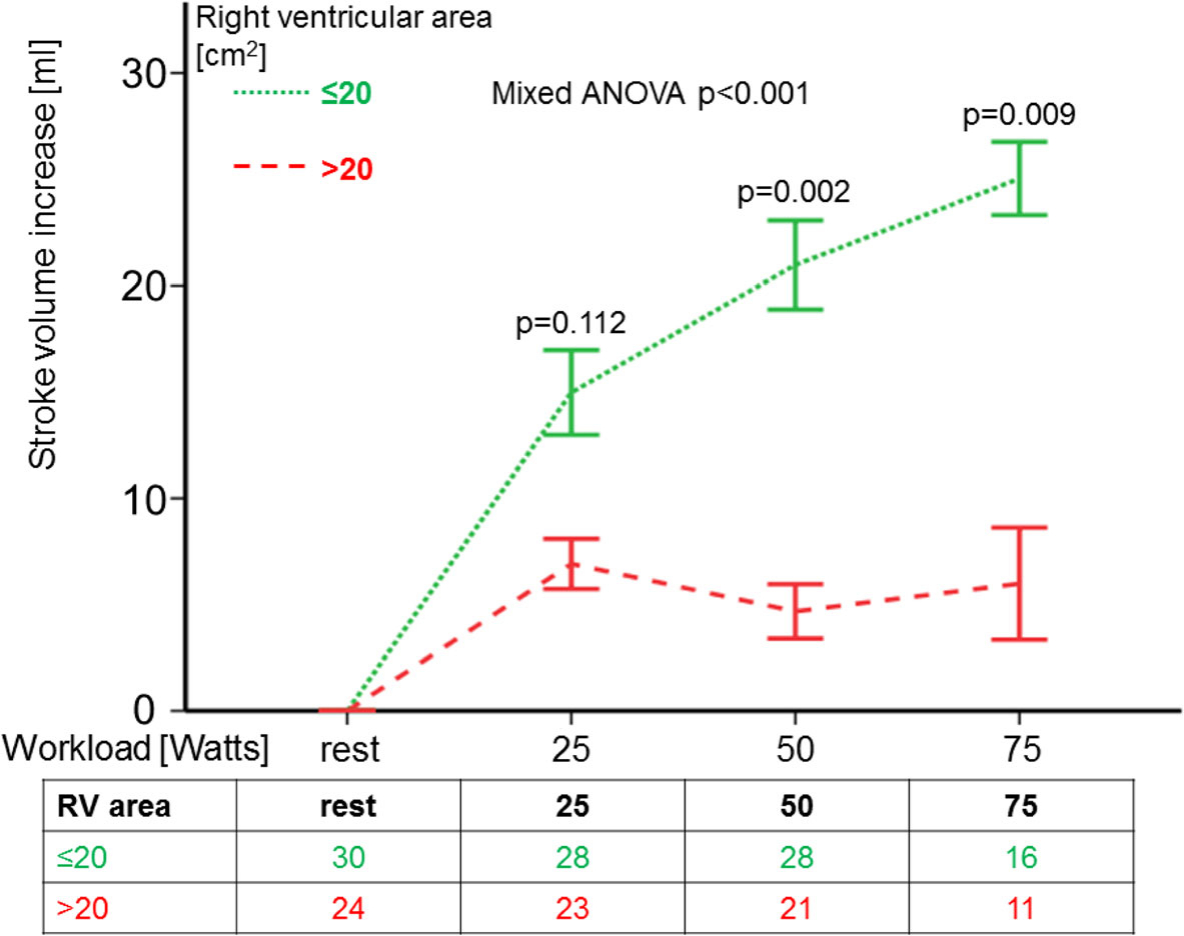
Course of stroke volume increase during exercise according to RV area. Patients with smaller (≤ median 20 cm^2^, dotted line) RV area showed significantly higher SV increase during exercise, than patients with larger RV area (> median 20 cm^2^; dashed line; mixed ANOVA p < 0.001). Bars indicate the standard errors of the mean difference

SV failed to increase in accordance with the exposed exercise level in patients with large RA- (*p* = 0.031) and/or RV area (*p* < 0.001; Fig. 3). Likewise, SVI was significantly higher in patients with small right heart size, compared to patients with enlarged RA and/or RV area (ANOVA RV p < 0.001, RA *p* = 0.001). Furthermore, patients with smaller RV, but not RA, presented with significantly higher peak PAC than patients with RV area above the median (39.5 ± 11.2 ml/mmHg vs. 33.2 ± 15.3 ml/mmHg, *p* = 0.027).

Sensitivity analysis with a threshold of 18 cm^2^ for RV area led to the same differences between groups with small and large right heart size. Furthermore, CI increase showed a statistically significant difference for each workload level.

**Fig. 4:**
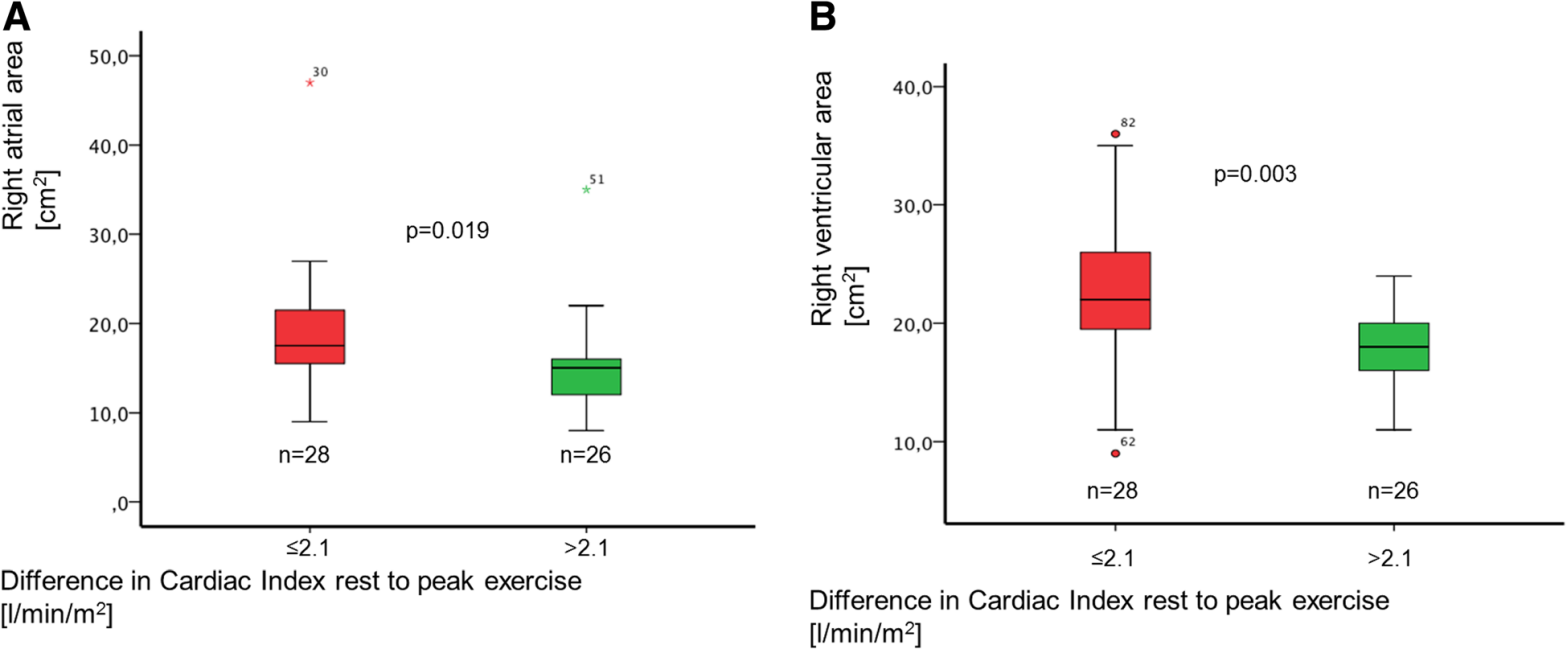
Difference of right heart size in patients with high and low ∆CI. Right heart size significantly differed between patients with high and low ∆CI_exercise_ according to the median of the complete sample of 2.1 L/min/m^2^ (**a**) RA area *p* = 0.019, (**b**) RV area *p* = 0.003; identical *p*-values for nonparametric and parametric testing)

When dichotomising the patient cohort according to RV output reserve (high and low ∆CI_exercise_) echocardiography showed considerable differences in RV and RA area (*p* = 0.003 and *p* = 0.019 respectively; Fig. 4a and b).

#### Factors associated with right heart size and RV output reserve (Tables 3 and 4)

##### Univariate analysis of right heart size and output reserve

**Figure 5:**
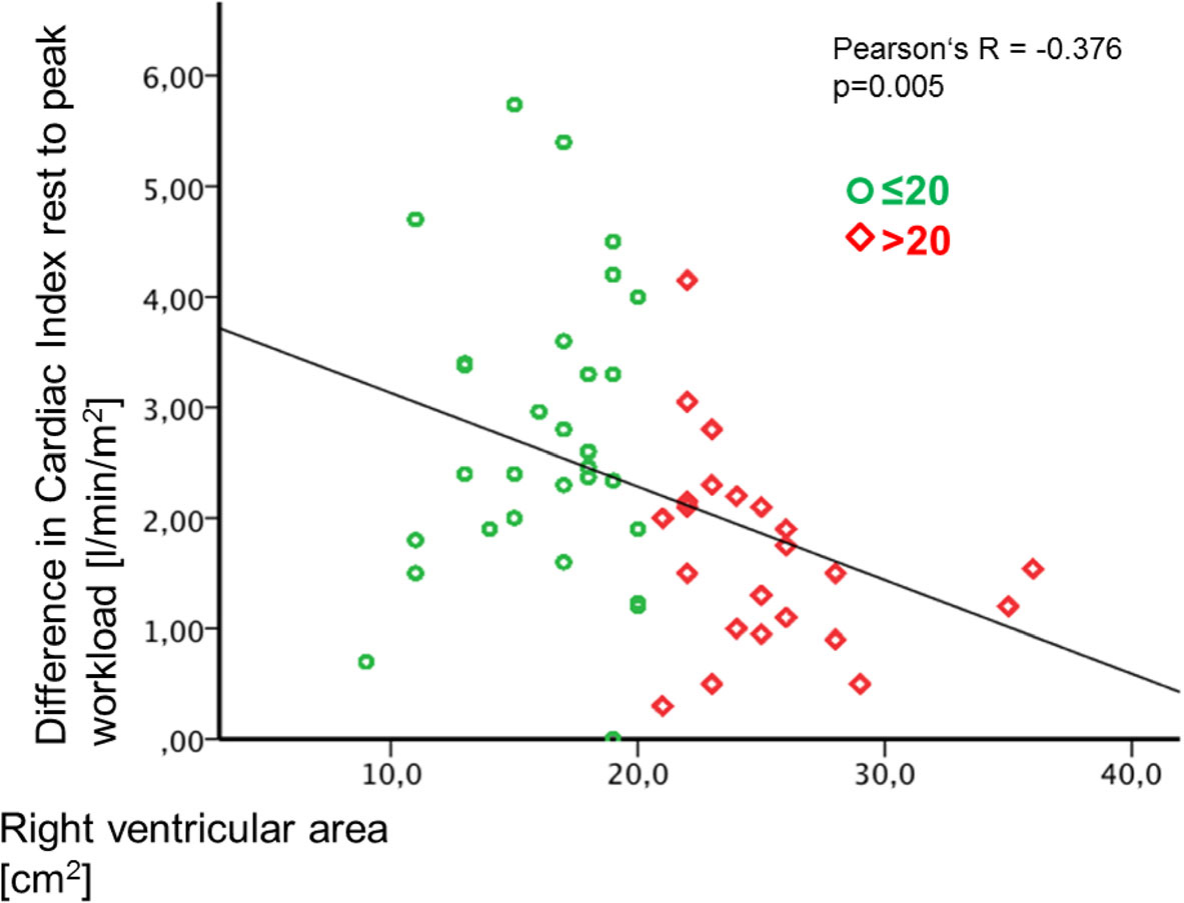
Correlation of RV-Area and ∆CI_**exercise.**_ RV area showed a significant negative correlation with ∆CI_exercise_ (*p* = 0.005)

In univariate regression analysis RV and RA area were significantly correlated with NT-proBNP, sPAP, CI during exercise, ∆CI_Peak_ (Fig. 5) and with right heart size (Table 3). RV area additionally significantly correlated with mPAP at rest, CI at rest, PVR at rest and peak mPAP.

**Table 3:** Correlation analysis of right heart size and clinical parameters

	Right atrial area				Right ventricular area			
	n	pearson’s R	p-value		n	pearson’s R	p-value	
Univariate analysis								
Age	54	0.193	0.162		54	- 0.048	0.209	
Body mass index	54	0.181	0.190		54	0.733	0.129	
6-min walking distance	53	- 0.108	0.441		53	- 0.121	0.387	
NT-proBNP	53	0.539	< 0.001	*	53	0.538	< 0.001	*
Echocardiography								
Systolic pulmonary arterial pressure	54	0.307	0.024	*	54	0.567	< 0.001	*
Right atrial area		–			54	0.703	< 0.001	*
Right ventricular area	54	0.703	< 0.001	*		–		
Tricuspid annular plane systolic excursion	54	- 0.128	0.356		54	- 0.082	0.554	
Cardiopulmonary exercise testing								
Peak oxygen consumption (V’O_2_)	54	0.042	0.736		54	0.051	0.713	
Peak oxygen consumption/kg (V’O_2_/kg)	54	- 0.199	0.149		54	- 0.227	0.099	
Right heart catheter								
rest								
Mean pulmonary arterial pressure	54	0.176	0.202		54	0.544	< 0.001	*
Cardiac Output	54	- 0.028	0.839		54	- 0.052	0.709	
Cardiac Index	54	- 0.209	0.129		54	- 0.281	0.040	*
Pulmonary arterial wedge pressure	54	0.025	0.857		54	- 0.101	0.467	
Pulmonary vascular resistance	54	0.175	0.206		54	0.508	< 0.001	*
Stroke volume index	54	−0.244	0.076		54	−0.301	0.027	*
exercise								
Mean pulmonary arterial pressure	54	0.097	0.486		54	0.419	0.002	*
Cardiac Output	54	- 0.177	0.200		54	- 0.223	0.104	
Cardiac Index	54	- 0.344	0.011	*	54	- 0.427	0.001	*
∆ CI peak	54	- 0.313	0.021	*	54	- 0.376	0.005	*
Stroke volume index	54	−0.264	0.054		54	−0.407	0.002	*
Lung function / Diffusing capacity								
DLCOc SB	48	- 0.052	0.723		48	- 0.003	0.982	
DLCOc /VA	48	0.176	0.231		48	0.137	0.352	

*CI* = Cardiac Index, *NT-proBNP* = N-terminal pro brain natriuretic peptide, *DLCOc SB* = diffusion capacity transfer factor, *DLCOc /VA* = diffusion capacity transfer coefficient

* = significant at level 0.05

**Table 4:** Uni- and multivariate regression analysis of RV output reserve

Univariate analysis (∆ CI _Peak_)	n	pearson’s R	*p*-value	
Age	54	0.424	0.001	*
Body mass index	54	0.092	0.506	
6 min walking distance	54	0.278	0.044	*
NT-proBNP	54	- 0.360	0.008	*
Echocardiography				
Systolic pulmonary arterial pressure	54	- 0.462	< 0.001	*
Right atrial area	54	- 0.313	0.021	*
Right ventricular area	54	- 0.376	0.005	*
Tricuspid annular plane systolic excursion	54	0.065	0.64	
Cardiopulmonary exercise testing				
peak oxygen consumption (V’O_2_)	54	0.466	< 0.001	*
peak oxygen consumption/kg (V’O_2_/kg)	54	0.380	0.005	*
Right heart catheter				
rest				
mean pulmonary arterial pressure	54	- 0.288	0.035	*
Cardiac Output	54	0.282	0.039	*
Cardiac Index	54	0.223	0.106	
Pulmonary arterial wedge pressure	54	- 0.016	0.906	
pulmonary vascular resistance	54	- 0.366	0.006	*
exercise				
mean pulmonary arterial pressure	54	- 0.073	0.598	
Cardiac Output	54	0.839	< 0.001	*
Cardiac Index	54	0.894	< 0.001	*
Lung function / Diffusing capacity				
DLCOc SB	48	0.361	0.012	*
DLCOc / VA	48	0.342	0.017	*
Multivariate analysis				
Logistic Regression (stepwise forward selection)				
∆ CI _exrcise (dichotomous)_		Exp (B)		
Right ventricular area	47	0.863	0.027	*
Linear Regression (stepwise forward selection)				
∆ CI _Peak (continuous)_		pearson’s R		
Right ventricular area	47	- 0.360	0.003	*
Age	47	- 0.412	0.001	*

*CI* = Cardiac Index, *NT-proBNP* = N-terminal pro brain natriuretic peptide, *DLCOc SB* = diffusing capacity transfer factor, *DLCOc / VA* = diffusing capacity transfer coefficient

* = significant at level 0.05, Exp (B) = Regression coefficient

∆CI_exercise_ significantly positively correlated with age, exercise capacity (6-MWD, peak oxygen consumption, peak oxygen consumption/kg/min), hemodynamics (CO at rest; peak CO and CI during exercise) and lung diffusing capacity (transfer factor DLCOc SB and transfer coefficient DLCOc / VA) (Table 4). A negative correlation was detected between ∆CI_exercise_ and NT-proBNP, echocardiographic parameters (sPAP, RA area, RV area) and hemodynamics at rest (mPAP, PVR).

##### Multivariate analysis of output reserve

Stepwise forward selection of multivariate logistic regression analysis showed RV area to be the single independent predictor for high or low ∆CI_Peak_ (regression coefficient 0.863, *p* = 0.027).

## Discussion

To the best of our knowledge this is the first study showing that in patents with PAH enlarged RV- or RA areas (measured by echocardiography) were associated with a significantly reduced RV pump function during exercise (lower ∆CI_exercise_) measured by right heart catheterization. Furthermore, the study revealed that PAH-patients with larger size of the right heart had higher pulmonary arterial pressures, pulmonary vascular resistance and NT-proBNP levels. Patients with higher RV-areas presented with a significantly lower stroke volume index and pulmonary arterial compliance at peak exercise than patients with smaller RV-size. RV area was identified as the only independent predictor of RV output reserve (lower ∆CI_exercise_). Thus, this study gives further evidence that assessing the right heart size by imaging techniques as echocardiography gives further important clues to RV pump function and cardiopulmonary hemodynamics.

### Right heart size, pump function

This study confirms the results of previous studies using MRI which showed that enlarged RV end systolic and end-diastolic volumes were obtained in patients with lower RV stroke volumes [12, 26]. However, in the first previous MRI-study RV volumes were not directly compared with pump function but with survival [26]. Large RV end-diastolic volume and SV at baseline were associated with poorer prognosis. Further dilation of RV with further decrease of SV during follow-up predicted a poor long-term outcome [26]. Most recently these findings have been confirmed by an analysis of the French PAH registry demonstrating that SVI and right atrial pressure were independently associated with death or lung transplantation at first follow-up after initial PAH treatment [27].

Our study demonstrates for the first time a negative relationship between right heart size and RV pump function using 2-D-echocardiography for assessing the RA- and RV-areas in the four chamber view and hemodynamic values from right heart catheterization at rest and during exercise. Patients with enlarged RV area had significantly lower CI and SVI at rest and during exercise. These patients had also higher mean pulmonary arterial pressure, pulmonary vascular resistance at rest and NT-proBNP levels which reflects a more severe disease. The negative impact of RV-enlargement was also demonstrated by a MRI-study which showed in patients with increasing RV volumes during follow-up a disease progression leading to death or transplantation whereas patients with stable RV volumes remained clinically stable [12]. Changes in RV volumes were even more sensitive parameters for deterioration than the repeated measurement of hemodynamics which remained unchanged [12]. In this study, patients with enlargement of RV volumes had a decline of RV ejection fraction [12]. Two further studies demonstrated a reduction in RV volumes by targeted PAH-therapy, which suggests an improvement of RV pump function [11, 28].

### RV output reserve and right heart size

RV output reserve, defined in this study as increase of CI during exercise measured by right heart catheterization, is an emerging parameter which has shown to be of prognostic importance in patients with PH [13, 14]. In this study RV area was identified as the only independent predictor of RV output reserve. This again shows that RV size may reflect the impairment of RV pump function. We hypothesize that increased PAC and reduced increase of RV output during exercise in patients with larger RV and/or RA areas, respectively is due to more severe pulmonary vascular disease. A both reproducible and clinically practical way to evaluate RV output reserve can be performed by invasive measurements^15^. Further prospective studies have to be conducted to evaluate the magnitude of the relation to right heart size and if non/invasive assessment of RA- and RV area or volume are useful for an estimation of RV output reserve.

Advanced PAH with increased pulmonary load leads to RV dilatation (heterometric adaptation) in order to maintain SV^6^. In this study RV output reserve was significantly linked to RV size.

### Study limitations

The retrospective, single-center design of this study with a rather small number of subjects limits the study results. A higher sample size may have led to identification of more independent factors in the multivariate analysis.

Echocardiographic assessments of the right heart are complicated by its complex shape and morphology. Especially in obese patients, patients with chest wall deformities or COPD, the correct assessment of RV size and function becomes a challenging task. In this respect, cardiac MRI becomes particularly appealing, as it does provide a thorough assessment of right heart size and function even in complicated conditions. In our cohort, high quality recordings were used and no comorbidities were interfering the test results. As the determination of RA and RV area is a readily available assessment which is practicable in a good quality, its application in clinical practice is more common compared to cardiac MRI. Unfortunately, no MRI data is available for this patient cohort to confirm the hemodynamic data.

Invasive measurements, cardiopulmonary exercise testing and echocardiographic parameters could not be assessed in one single examination. In order to reduce the influence of inter-exam variations, we only included patients that underwent all measures within a time frame of 48 h. The assessment of CI may be complicated by tricuspid insufficiency in patients with PH. Due to the bidirectional blood flow through the tricuspid valve CI may be overestimated in some patients, which may have influenced the results.

The correlation of right heart size and function to TAPSE and other parameters and their prognostic value should be investigated in a larger-scale study.

## Conclusion

The study shows that assessment of right heart size is important for RV functional characterization and may be helpful since it reflects RV pump function and RV output reserve. RV and RA area by 2-D echocardiography represented a valuable and easily accessible indicator of RV pump function at rest and during exercise. Therefore, these results may be relevant for clinical practice. RV output reserve should be considered as an important clinical parameter. However, prospective studies are needed for further evaluation.

## Abbreviations

∆: Difference
6MWD: 6-min walking distance
ANOVA: Analysis of variance
APAH: Associated PAH
BMI: Body Mass Index
CI: Cardiac Index
CO: Cardiac Output
CTEPH: Chronic thromboembolic pulmonary hypertension
DLCOc / VA: Diffusing capacity transfer coefficient
DLCOc SB: Diffusing capacity transfer factor
dPAP: Diastolic PAP
Exp (B): Regression coefficient
HPAH: Heritable PAH
HR: Heart rate
IPAH: Idiopathic pulmonary arterial hypertension
mPAP: Mean pulmonary arterial pressure
MRI: Magnetic resonance imaging
NT-proBNP: N-terminal pro brain natriuretic peptide
PAC: Pulmonary arterial compliance
PAH: Pulmonary arterial hypertension
PAP: Pulmonary arterial pressure
PASP: Pulmonary arterial systolic pressure
PCWP: Pulmonary capillary wedge pressure
PH: Pulmonary hypertension
PVR: Pulmonary vascular resistance
RA: Right atrial
RHC: Right heart catheter
RV: Right ventricular
sPAP: Systolic PAP
SV: Stroke volume
SVI: Stroke volume index
TAPSE: Tricuspid annular plane systolic excursion
TRV: Tricuspid regurgitation velocity
TTE: Transthoracic echocardiography
VO’_2_: Oxygen consumption
